# Physiologically-Relevant Cellular Models for Biomarkers and Drug Discovery

**Published:** 2018-04-26

**Authors:** Araceli Espinosa Jeffrey

**Affiliations:** Department of Neurobiology, Psychiatry and Biobehavioral Sciences, David Geffen School of Medicine at UCLA, USA

## Short Communication

**Figure 1:**
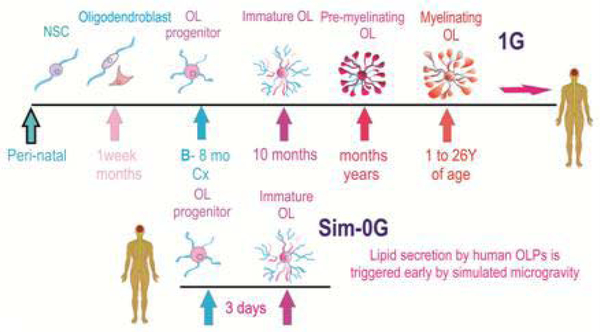
Detailed knowledge of the chronology of lipid synthesis by HuOLs has been only partially elucidated. We have recently reported an accelerated secretion of membrane-forming lipids by HuOLPs exposed 3 days to sim-0G. This was not the case in OLPs that were maintained in 1G (Adapted from Espinosa-Jeffrey et al., 2016, with permission).

Dr. Peter Stern, senior editor at Science, recently wrote that “these are exciting times for neuroscience” and I could not agree more because, for the first time, one of the big questions in our field may be answered soon: “Where are the cures?” with the advent of Human Induced Pluripotent Stem cells (hiPS), “cures” for Central Nervous System (CNS) ailments appear to be around the corner. Moreover, at present, global efforts to establish biological research in outer space to help humanity worldwide are increasing, the goals to be ascertained need to be precise as well as immediately addressed, as astronauts who help performing science in the International Space Station, either in space or upon their return to earth, have been diagnosed with chronic intracranial hypertension after 34 cumulative days. Assessments on astronauts’ health while in space are being collected and the variations acknowledged. However, this kind of data may lead to a slow, unclear understanding of the causes for the variations found, which might not be the most prominent or health-threatening. On the other hand, studying brain cells at the cellular and molecular level is of outmost importance for astronauts’ well-being. A complete understanding on the pathophysiology of chronic intracranial hypertension in astronauts remains elusive; however, it could be associated with unforeseen aspects related with brain biology. Based on previous reports from other scientists and our own work performed on earth through 35 years (in normal gravity = 1G) and using the available data as a bench mark, we have pioneered the study on the impact of simulated microgravity (sim-0G) on neural stem cells and oligodendrocytes (OLs). Using a homogeneous cell culture approach containing only neural stem cells or OLs we found increased neural stem cell proliferation induced by sim-0G [1]. OL-progenitors (OLPs) also proliferate more insim-0G with a concomitant shortening of their cell cycle [2]. During development in 1G this population is extensive in the post-natal brain and in restricted numbers representing 5% of quiescent OLs in the adult brain. Nonetheless, they might be responding in the same manner in “real microgravity” in space, increasing the number of OLPs in the brain of astronauts. Fortunately, we may get information on the effects of real microgravity on OLPs, more mature OLs and neural stem cells soon, as our cells will be flying to space sometime in 2018. While waiting for the space flight and in an attempt to understand better how microgravity impacts other aspects of OLs biology, we have analyzed the secretome of human post-mitotic OLs in sim-0G, this study led us to the surprising discovery on the accelerated and enhanced secretion of fatty acids, membrane forming lipids and complex lipids by HuOLs after a three-day exposure to sim-0G, a phenomenon not seen in cells kept in 1G as seen in (Figure 1) [3]. In rodents, myelination occurs predominantly during the first month of life [4]. In humans it starts pre-natally and continues postnatally. It has been estimated that the myelin-membrane surface area of one OL expands at a rate of 5–50 × 103 μm^2^/day, compared with the surface area of the cell soma (i.e., the plasma membrane) of 0.3 × 103 μm^2^ [5], around 6,500-fold increase in membrane surface between an immature and a fully myelinated OL [6]. In humans there is brain region heterogeneity for myelination and therefore such window is still being defined. Nonetheless, if one takes the presence of OLPs and “preOL” together with the fact that White Mater Injury (WMI) in the developing human brain peaks in incidence around 28 to 32 weeks post-conception in white matter regions, we can say that myelination starts prenatally and continues from months to years after birth. Taken together, increased cell proliferation as well as the enhanced lipid-secretion by OLs while in microgravity, suggest that these two phenomena may contribute to chronic intracranial hypertension reported in astronauts. Nonetheless, for human kind on earth these may mean great news as on one hand, one can increase OLs numbers *via* proliferation and on the other, these cells could be primed by a short exposure to simulated microgravity followed by transplantation into the myelin deficient brain or spinal cord. There is also evidence that oligodendroglial tumors constitute between 5 and 20 percent of all glial tumors but, despite the prolonged clinical course seen with oligodendroglial tumors, the prognosis is almost always fatal. Therefore, understanding the gravitational modulation of OLPs’ intrinsic plasticity to increase their progenies might also hold the key to treat oligodendroglial tumors. This is the beginning of an extensive study on the impact of microgravity in the CNS; therefore, we have started studying one cell type at a time, prior to studying co-cultures such as neurons and OLs aiming at myelination in microgravity, or with endothelial cells and astrocytes to understand cell-cell interactions and even discover molecules contained in the secretome of these co-cultures. From our study we believe that microgravity holds the key to: 1) Myelin regeneration and repair; as well as 2) modulation of neural cell proliferation. Based on our data from simulated microgravity, we are confident that we will unveil specific molecule(s) i.e. biomarkers, pathways and/or receptors that may be implicated in increased cell proliferation as the first step to address modulation of neural cell proliferation while in space in health and disease. Thus, funding and sustained timely efforts should be focused on an intense study of these and other changes elicited by microgravity in Central Nervous System (CNS) cells. Only after having thoroughly performed the pertinent studies, we shall be able to discover applicable, successful actions to counteract any danger or threat for astronauts returning from space, as well as to harness effective, preventive methods and treatments, prior to or during a long-term space flight. As an added value to our scientific social responsibility, we strongly believe that the outcome of this branch of our research will enable the scientific community to establish the course of action and accurate procedures to permeate our scientific knowledge to find effective, cures for CNS ailments that afflict mankind on earth.
